# Laser-assisted guiding of electric discharges around objects

**DOI:** 10.1126/sciadv.1400111

**Published:** 2015-06-19

**Authors:** Matteo Clerici, Yi Hu, Philippe Lassonde, Carles Milián, Arnaud Couairon, Demetrios N. Christodoulides, Zhigang Chen, Luca Razzari, François Vidal, François Légaré, Daniele Faccio, Roberto Morandotti

**Affiliations:** 1 Institut National de la Recherche Scientifique–Énergie Matériaux Télécommunications, 1650 Boulevard Lionel-Boulet, Varennes, Québec J3X 1S2, Canada.; 2 School of Engineering and Physical Sciences, Scottish Universities Physics Alliance, Heriot-Watt University, Edinburgh EH14 4AS, UK.; 3 The MOE Key Laboratory of Weak Light Nonlinear Photonics, School of Physics and TEDA Applied Physics School, Nankai University, Tianjin 300457, China.; 4 Centre de Physique Théorique CNRS, École Polytechnique, F-91128 Palaiseau, France.; 5 College of Optics, Center for Research and Education in Optics and Lasers, University of Central Florida, Orlando, FL 32816, USA.; 6 Department of Physics and Astronomy, San Francisco State University, San Francisco, CA 94132, USA.; 7 Institute of Fundamental and Frontier Sciences, University of Electronic Science and Technology of China, Chengdu 610054, China.

**Keywords:** Laser ionization, Electric discharges, Beam shaping, Plasma

## Abstract

Electric breakdown in air occurs for electric fields exceeding 34 kV/cm and results in a large current surge that propagates along unpredictable trajectories. Guiding such currents across specific paths in a controllable manner could allow protection against lightning strikes and high-voltage capacitor discharges. Such capabilities can be used for delivering charge to specific targets, for electronic jamming, or for applications associated with electric welding and machining. We show that judiciously shaped laser radiation can be effectively used to manipulate the discharge along a complex path and to produce electric discharges that unfold along a predefined trajectory. Remarkably, such laser-induced arcing can even circumvent an object that completely occludes the line of sight.

## Teaser

We demonstrate that laser beam shaping can be used to precisely control an electric discharge trail, avoiding or bypassing obstacles in the line of sight.

## INTRODUCTION

Since the dawn of civilization, electric discharge phenomena such as lightning have played a key role in the scientific understanding of electricity itself. Today, these same processes are an integral part of modern technologies and find applications in numerous settings. In gases, they typically occur when the applied voltage between two electrodes establishes a field that exceeds breakdown, which in air is about 34 kV/cm under standard conditions for temperature and pressure. In manufacturing, arc discharges are routinely used for machining (*1*) and micromachining (*2*), and for assisting the milling process (*3*). They are also used for fuel ignition in combustion engines (*4*–*6*) as a means to control the hydrodynamics of high-speed gases (*5*, *6*), and could ultimately be used in monitoring and controlling pollution (*7*, *8*). Yet, despite such advances, developing methods to effectively control and shape the path of an electrical spark along a predetermined trajectory still remains a significant challenge.

**Fig. 1:**
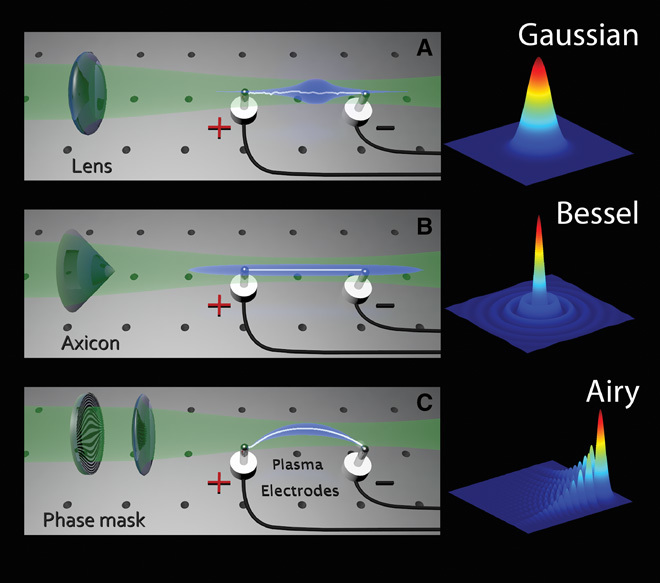
Laser-guided discharges. (Left panels) Electric discharges resulting from different beam shaping configurations. (**A** to **C**) These include a standard Gaussian beam focused by a lens (A), a Bessel beam formed after an axicon (B), and an Airy beam produced by a binary phase mask (C). The laser is represented in green, the plasma channel is in blue, and the discharge is depicted in white. (Right panels) Intensity distribution corresponding to the different beam shaping configurations.

**Fig. 2:**
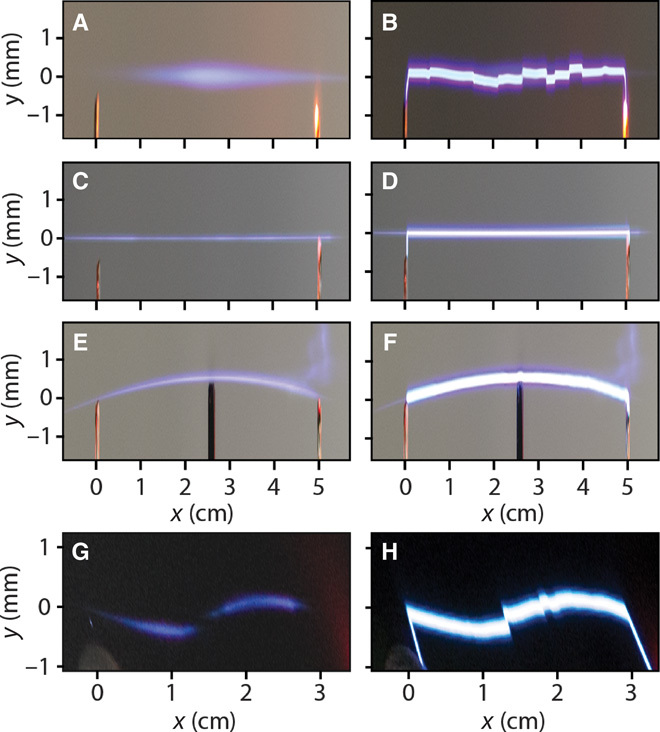
Shaped laser plasmas and electric discharges. Different shapes associated to the discharges that can be achieved through judicious beam shaping. (**A** and **B**) Gaussian beam. (**C** and **D**) Bessel beam. (**E** and **F**) Airy beam. (**G** and **H**) S-shaped beam obtained by properly combining two conventional Airy beams. (A, C, E, and G) Photographs taken when no voltage is applied (showing the laser beam path through ionization-induced fluorescence). (B, D, F, and H) Discharge in the presence of a high voltage between the two electrodes.

It will be of fundamental importance to devise schemes through which arcing can be fully controlled. Here, we show that properly shaped laser beams can provide a mean to this end by virtue of the ionization they induce in air (see Fig. 1) [for earlier works on laser-assisted electric discharges, see (*9*–*13*), and for the latest investigations, see (*14*, *15*)]. The recent introduction of self-bending Airy beams in optics (Fig. 1C) has opened up new opportunities in terms of propagating spatially accelerating wavefronts along complex curved trajectories (*16*), and we demonstrate that it can also provide a new degree of freedom in controlling electric discharges. By manipulating the specific shape of a laser beam, it is indeed possible to precisely control the trail of a spark. Along these lines, parabola-like and S-shaped electrical discharges can be achieved (see Figs. 1 and 2), thus transporting the electrical charge around objects that would have otherwise completely blocked the discharge itself. Another important attribute of an Airy beam is its ability to spontaneously reform its main intensity features after encountering an obstacle. This property, referred to as self-healing, is shared also by another important class of subdiffractive beams, the so-called Bessel beams (Fig. 1B) (*17*). Remarkably, we show that the self-healing properties of Airy and Bessel laser wavefronts can be readily transferred to the electrical discharge, which also self-heals and resumes its original trajectory even after a direct hit on an obstacle. In this regard, not only is the unpredictability that usually accompanies this phenomenon removed, but also the discharge can be manipulated in such a way that the resulting arc can bypass an object placed in the line of sight between the two electrodes.

## RESULTS

As a reference, we first consider the case where a standard Gaussian beam guides the discharge (as illustrated in Fig. 1A). We focused the Gaussian beam delivered by an amplified Ti:sapphire system with a lens (focal length *f* = 100 cm) between two wire electrodes. The input energy is 15 mJ, the beam full width at half maximum (FWHM) is 10 mm, and the pulse duration is 50 fs. After an optical filament is formed (*18*), the laser pulse ionizes the air between the electrodes, as can be seen from the resulting fluorescence shown in Fig. 2A, and deposits energy that induces an expanding heat wave. In turn, the heated air column locally reduces the gas density, thus lowering the breakdown voltage over a path defined by the laser-induced ionization (*19*, *20*). The electron avalanche process depends on *E*/*N*, where *E* and *N* are the local electric field strength and air density, respectively (*21*). When a relatively high voltage is applied (nearly 15 kV over a 5-cm gap—a field far lower than that for the air breakdown at 1 atm), a discharge occurs between the electrodes, and it follows the path of the least resistance, that is, along the trajectory determined by the laser beam (see Fig. 2B). Note that in this case, the arc trail is heavily distorted and effectively unpredictable. In Fig. 2 (C and D), we present the case of a discharge triggered and guided by a Bessel beam with a 4.6° cone angle as obtained by means of a fused silica axicon with a 10° base angle for electrode distance and an applied voltage equal to the previous case. The central high-intensity peak of the subdiffractive Bessel beam has a diameter of ~7 μm and is thus much smaller than the diameter of the Gaussian optical filament (~50 μm). As a result, plasma excitation is more localized in the transverse direction (Fig. 2C), and the electric discharge (Fig. 2D) is thus guided along a much better defined path, with no evidence of random jumps as seen in Fig. 2B. We then repeat this same experiment (that is, for the same voltage and electrode distance) with a self-bending beam that is generated using an aberrated cylindrical lens system (*22*). This beam propagates along a curved parabolic trajectory with a subdiffractive intensity peak of nearly 20-μm diameter. As shown in Fig. 2E, the induced plasma follows the curved excitation by the laser pulse (*23*) [this is also the case for plasmonic Airy and Bessel beams (*24*, *25*)], and we observe that the electric discharge also occurs along the same curved path (see Fig. 2F). As presented in the figure, the self-bending beam is capable of creating a plasma channel that circumvents an obstacle (the edge of an opaque glass) that would have otherwise blocked the direct line of sight between the two electrodes. That is, the discharge follows the plasma trajectory and thus travels around the obstacle without damaging (via corona discharge) the object itself. Finally, more complex arc shapes can be easily realized. For example, by using a binary phase mask that transforms the input laser pulse into two concatenated Airy beams, an S-shaped plasma channel is produced as shown in Fig. 2G. Also in this case, the electric discharge is guided along the preassigned path (Fig. 2H).

We further investigated the electric breakdown for the three cases shown in Fig. 1. We observed a significant reduction of the breakdown field (compared to the standard unguided configuration) that is 3.5 and 10 times lower for the cases of the self-bending and of the Bessel beam, respectively (see the Supplementary Materials). Therefore, we estimate that a guided discharge may be supported along any arbitrary trajectory [see, for example, (*26*–*30*)] that has a ratio of at most 3.5 between the arc length and the interelectrode distance.

**Fig. 3:**
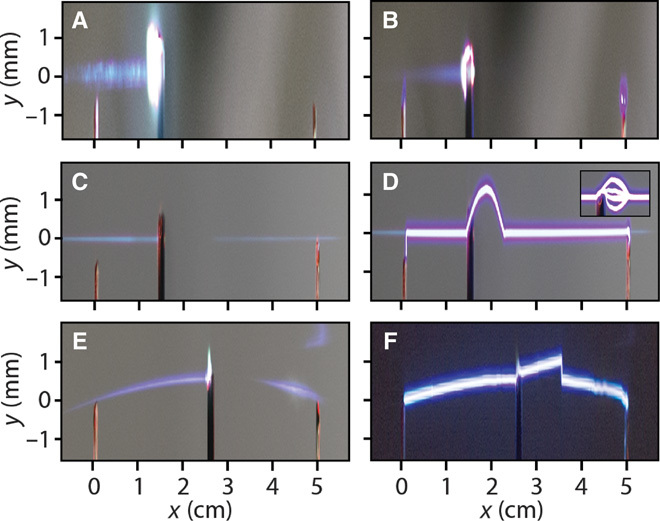
Effect of an obstacle placed in the control beam path. (**A**), (**C**), and (**E**) show how the intense part of the beam (the blue fluorescence in the photographs) is modified upon the insertion of an object in the beam path for a Gaussian, a Bessel, and an Airy beam, respectively. In the Gaussian case, the intensity of the beam is completely quenched after the obstacle and there is no way to guide an electric discharge between the two poles shown in (**B**). (**D** and **F**) In contrast, for both the cases of a Bessel-type and of an Airy-type propagation, the beam restores itself after the obstacle and the electric discharge occurs along an almost unaffected trajectory. The inset in (D) shows a multishot acquisition where the discharge in the region right after the obstacle chooses different paths for different shots, before converging to a straight trajectory when self-healing takes place.

We then perform experiments where an obstacle is placed directly in the beam path so that it completely blocks the main intensity peak of the laser pulse. For the case of a standard Gaussian beam, this results in a complete quenching of the beam, and no light is transmitted. As shown in Fig. 3A, the conductive channel is also obstructed, and no discharge occurs when the potential is applied between the two electrodes (Fig. 3B). The situation is completely different in the case of nondiffracting laser beams. Indeed, for such waveforms, it has been demonstrated that even if the main intensity peak is blocked while the remaining part of the beam is allowed to pass, self-healing takes place (*31*–*33*) and the intense part of the beam reconstructs itself after encountering an obstacle. This can be clearly seen in Fig. 3 (C and E), where the plasma fluorescence generated by a Bessel and an Airy beam, respectively, reappears after few millimeters, beyond an opaque screen that is placed in the beam path. As a consequence, we observe that the electric discharge channel can also self-heal so that it may propagate between the two electrodes (see Fig. 3, D and F). We note that in the region immediately after the obstacle, where the laser beam has not healed yet and is thus not able to substantially ionize the medium, the electric path is actually random from one laser shot to the next (see inset in Fig. 3D). However, it is the presence of the healed plasma channel after the obstacle that ensures that the discharge actually occurs and follows a well-defined path along the optical beam trajectory. This result also holds in the case of an isolated conductive obstacle (see the Supplementary Materials).

A deeper understanding of the complex physics associated with the discharge self-healing process can be obtained by considering numerical models for describing the optical spatiotemporal dynamics as well as the onset of the electric arc. The numerical solution to this problem is obtained in a three-step procedure (see the Supplementary Materials for more details), consisting of (i) the simulation of the nonlinear pulse propagation that “deposits” energy in the gas via field ionization (neglecting collisional processes) (*19*, *34*), (ii) the simulation of the gas hydrodynamics under nonequilibrium conditions resulting from the heating associated to the plasma recombination (*35*, *36*), and, finally (iii) the simulation of the electric discharge that takes place along the lower gas density channel determined by the gas hydrodynamics (*37*). We note that the electric discharge process does not interfere with the creation of the plasma induced by the focused laser because it occurs several nanoseconds after the laser pulse passage. As an example, we focused our attention on the case of an object placed in the path of a diffraction-free Bessel beam.

**Fig. 4:**
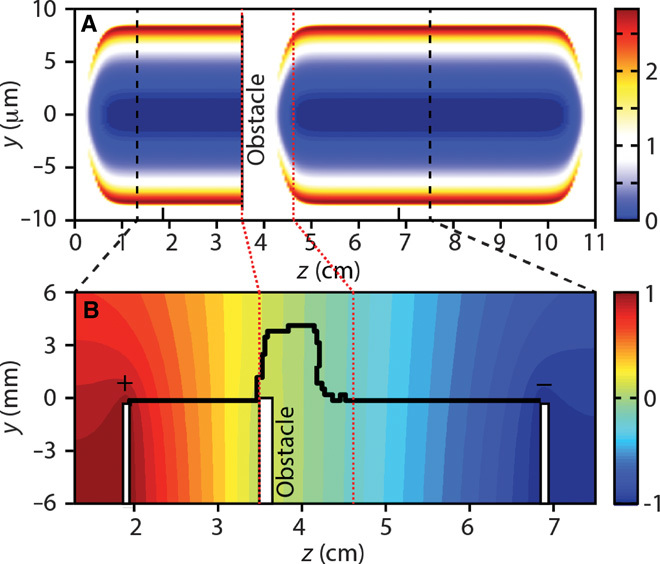
Air density profile. (**A** and **B**) Simulation of the air density profile normalized to the ambient air density for the Bessel beam after 6.5 ns (A) and the corresponding discharge path (B). The colormap in (B) represents the initial normalized electric potential. Note that (B) shows a rescaled section of (A).

We simulated the pulse propagation using parameters that are compatible to those encountered in our experiment (*18*). The gas temperature increase (*T*) induced by the laser pulses on the gas medium is estimated from the observed breakup voltage by means of the Paschen law, as detailed in the Supplementary Materials. In the case of a Bessel beam and for the parameters used in our experiment, beam dynamics leads to a local temperature rise of nearly 50,000 K. Using this temperature increase as an input condition, we simulated the gas hydrodynamics and we found that a large density depression is established on axis after 6.5 ns and that the gas density decreases to 7% of the normal gas density. Figure 4A shows the air density map obtained via this hydrodynamic simulation considering a 2-mm-width stopper in the beam path. We then simulated the discharge propagation using a stochastic model similar to that used by Niemeyer *et al*. (*37*). To do so, we assumed a growth probability *P*_*i*_ ≅ (*E*_*i*_/*N*_*i*_)^5^/∑_*j*_(*E*_*j*_/*N*_*j*_)^5^, where the index *i* denotes the possible leader growth directions at a given time step. Here, the electric field *E*_*i*_ is obtained by solving the two-dimensional Laplace equation for the electric potential, whereas the air density *N*_*i*_ is determined from the hydrodynamic simulation (Fig. 4A). In this model, leaders start simultaneously from both electrodes when *E*_*i*_/*N*_*i*_ is greater than some threshold values, and meet in between to bridge the air gap. The simulation (Fig. 4B) shows that the discharge closely follows the rectilinear air-depleted path left by the laser while it is wandering with a few millimeters deviation from the straight trajectory in the ~1-cm region past the dielectric stopper, where the Bessel beam is partly quenched. These predictions are in agreement with our experimental results.

We note that the discharge self-healing process is restrained by the intrinsic limitations of the optical self-healing and also by the requirement that the available potential difference at the electrodes is sufficient to generate a field higher than that of the breakdown across the broken plasma channel. For example, in the case of the Bessel beam investigated here, our 35-kV dc source will discharge over a maximum distance of the order of 1 cm, corresponding to an obstacle width of ~1.6 mm (that determines the distance over which the Bessel channel self-heals). This prediction is compatible with our experimental observations. If we upscale the dc potential, for example, to 1 MV and also the laser power/optical element aperture to extend the length of the laser ionized channel, we may speculate that the maximum allowed obstacle width could scale to ~5 cm.

## DISCUSSION

The proposed technology paves the way to the systematic and precise control of a high-voltage discharge along predetermined paths and provides a new degree of freedom in controlling electrical discharge–driven phenomena, opening an array of possibilities for both scientific and technological settings. We have shown that Airy and Bessel beams allow the discharge to circumvent obstacles, to propagate along a defined path (avoiding an erratic, lightening-like trajectory), and to self-heal in the presence of an obstacle. Other beam geometries, such as nonparaxial accelerating beams (*26*–*30*) and plasma waveguide arrays (*38*), may play an essential role in the further development of this new and powerful degree of control. For example, two skewed Bessel beams may be used to trigger an arc in gas at a remote location, whereas other configurations (such as abruptly autofocusing beams) can be used to induce arcing with very high spatial accuracy. The development of reconfigurable (telescopic like) systems of refractive or holographic optics that can dynamically control both the beam trajectories and bending angles of optical pulses with intensities sufficient to induce air ionization in the region of interest will be instrumental for the technological impact of the proposed ideas.

Finally, in a two-pulse excitation scheme, the plasma length is enhanced by properly choosing the delay between the pulses, because of the response associated with the molecular alignment (*39*), and may thus be considered as a further degree of freedom to control laser-guided electric discharges.

## MATERIALS AND METHODS

We have modeled the experimental observation of laser-guided electric discharge, with or without an obstacle in the beam path, by considering, without loss of generality, the case of a Bessel beam. A first step consists in computing the nonlinear propagation of the optical pulse generated by our laser source in air, accounting for the space-time effects as detailed in the Supplementary Materials. By considering a Gaussian pulse with an FWHM of 50 fs, an energy of 15 mJ, a beam width of 1-cm FWHM, and an 800-nm wavelength, focused by an axicon with a cone angle of 4.6°, we observed that the peak intensity of the pulse reaches nearly 10^14^ W/cm^2^ and remains fairly constant over more than 10 cm, thus ionizing the totality of oxygen and nitrogen molecules. We also investigated the dynamics of the laser-induced ions and free carriers subsequent to the laser passage. Following the model proposed by Zhao *et al*. (*40*), we found that fractions of a nanosecond after the pulse transits, electron-ion recombination nearly neutralizes the medium. We hence simulated the hydrodynamic expansion of heated air by solving the compressible fluid Euler equations in two-dimensional cylindrical coordinates. The integration of the fluid equations is carried out through the Harten Lax van Leer first-order Godunov method with the restoration of the contact wave (HLLC) (*35*). The system of equations is closed by means of the equation of state and the internal energy in the ideal gas form. The initial condition in the simulation consists of a temperature distribution that follows from the shape of the plasma generated by the laser. More specifically, we considered the first lobe of a Bessel function interrupted by a stopper and self-healed, as can be deduced by observing the fluorescence in Fig. 3C of the main text. For more details on the model and on the self-healing length, see the Supplementary Materials. The gas temperature was evaluated considering the Paschen law (*V*_B_ ∝ ρ, where *V*_B_ is the breakdown voltage and ρ is the gas density) together with the observed experimental decrease of *V*_B_ by an order of magnitude with respect to the case of unheated air and for the Bessel beam case. In the Supplementary Materials, we provide a detailed discussion on the experimental evaluation of this factor for three beam profiles (Gaussian, self-bending, and Bessel). This leads to a temperature increase of the order of Δ*T* ≈ 50,000 K. To model the discharge evolution between the two electrodes, we adopted a stochastic model similar to that of Niemeyer *et al*. (*37*). A detailed account of the simulation parameters that provide results in agreement with the experimental observations is given in the Supplementary Materials.

## Supplementary Material

http://advances.sciencemag.org/cgi/content/full/1/5/e1400111/DC1

## SUPPLEMENTARY MATERIALS

Supplementary material for this article is available at http://advances.sciencemag.org/cgi/content/full/1/5/e1400111/DC1 Text Fig. S1. Pulse propagation simulations. Fig. S2. Density profiles. Fig. S3. Additional experimental results. References (*41*, *42*)
