# Supplementary material for: Laser-assisted guiding of electric discharges around objects
Source: Sci Adv. 2015 Jun 19;1(5):e1400111. doi: 10.1126/sciadv.1400111 (PMC4640611; doi:10.1126/sciadv.1400111)
Supplement: http://advances.sciencemag.org/cgi/content/full/1/5/e1400111/DC1 [file supp_1_5_e1400111__index.html]

Science Advances | Science Advances

## Supplementary Materials

**This PDF file includes:**

- Text
- Fig. S1. Pulse propagation simulations.
- Fig. S2. Density profiles.
- Fig. S3. Additional experimental results.
- References (*41, 42*)

Download PDF

**Files in this Data Supplement:**

- Adobe PDF - 1400111\_SM.pdf
