# Supplementary material for: Laser-assisted guiding of electric discharges around objects
Source: Sci Adv. 2015 Jun 19;1(5):e1400111. doi: 10.1126/sciadv.1400111 (PMC4640611; doi:10.1126/sciadv.1400111)
Supplement: http://advances.sciencemag.org/cgi/content/full/1/5/e1400111/DC1 [file 1400111_SM.pdf]

Science Advances

www.advances.sciencemag.org/cgi/content/full/1/5/e1400111/DC1

Supplementary Materials for

Laser-assisted guiding of electric discharges around objects

Matteo Clerici, Yi Hu, Philippe Lassonde, Carles Milián, Arnaud Couairon, Demetrios N. Christodoulides, Zhigang Chen, Luca Razzari, François Vidal, François Légaré, Daniele Faccio, Roberto Morandotti

Published 19 June 2015, *Sci. Adv.* **1**, e1400111 (2015)
DOI: 10.1126/sciadv.1400111

This PDF file includes:

Text
Fig. S1. Pulse propagation simulations.
Fig. S2. Density profiles.
Fig. S3. Additional experimental results.
References (*41*, *42*)

Supplementary Text

The supplementary material reports a detailed description of the numerical model (Section 1), and provides additional data and a further description of the experiment (Section 2).

In particular (Section 1), we first computed the nonlinear propagation dynamics of the driving optical pulse in air (see Sec.1.1, where we considered, e.g., the Bessel beam case), and then evaluated the properties of the excited plasma in a DC bias field that led to the heating of air (Sec. 1.2 and 1.3). Once this dynamics was established, we numerically described the evolution of the gas density and determined the conditions for which a conductive, low-density channel was generated (Sec. 1.4). Finally, we simulated the behavior of the electric discharge guided in the conductive channel (Sec. 1.5).

In the experimental part (Sec. 2) we describe in more details the specific conditions in which the experiments have been performed and we provide additional data that support and complement the conclusions reported in the main text.

1. Numerical Model

1.1 Spatio-temporal modeling of the ionizing optical Bessel beam

The propagation of the spatio-temporal electric field envelope, $E(r,t,z)$, is assumed to obey the forward Maxwell equation written in the frequency domain for $\widehat{E}(r,\omega,z)$ as:

$$\partial_z \widehat{E} = i\left[k(\omega) - k_0 - k_0'(\omega-\omega_0)\right]\widehat{E} + \frac{i}{2k(\omega)}\Delta_\perp \widehat{E} + \frac{i}{2\varepsilon_0 c n(\omega)}\left(\omega\widehat{P} + i\widehat{J}\right), \qquad (1)$$

where the dispersion is described by the Peck & Reeder relation for the refractive index of air $n(\omega)$ *(40)*, and the propagation constant is $k(\omega) \equiv \omega n(\omega)/c$. In a cylindrical

geometry with revolution symmetry, the diffraction operator reads: $\Delta_\perp \equiv \left(\partial_r^2 + \frac{\partial_r}{r}\right)$. The nonlinear polarization $P(r,t,z)$ and current $J(r,t,z)$ describe the optical Kerr and plasma effects, including the nonlinear absorption of energy as well as the change of refractive index:

$$P(r,t,z) = 2\varepsilon_0 n_0 n_2 I E$$
$$J(r,t,z) = \varepsilon_0 n_0 c\left[\sum_l \left\{\beta_{K_l} I^{K_l-1}\frac{N_l}{N_l^{(0)}}\right\} + \sigma\left(1+i\omega_0\tau_c\right)N_e\right]E \quad ,$$

where $I \equiv \varepsilon_0 n_0 c|E|^2/2$ denotes the pulse intensity, N_e denotes the electron density and $l \equiv O_2, N_2$ stands for the molecule species in air. Eq. (1) is coupled to the rate equations for the density of ionized oxygen and nitrogen molecules in air:

$$\partial_t N_{O_2^+} = \sigma_8 I^8\left(N_{O_2}^{(0)} - N_{O_2^+}\right) + \frac{0.21\sigma I}{U_{i,O_2}}N_e - \beta_{e-O_2^+}N_e N_{O_2^+}$$
$$\partial_t N_{N_2^+} = \sigma_{11} I^{11}\left(N_{N_2}^{(0)} - N_{N_2^+}\right) + \frac{0.79\sigma I}{U_{i,N_2}}N_e - \beta_{e-N_2^+}N_e N_{N_2^+} \quad . \qquad \text{(2a,b)}$$

where the first term in Eqs. 2a and 2b describes multiphoton ionization, the second term describes collisional ionization and the third term describes electron-ion recombination. The electron plasma number density is obtained from the densities of the positive ions, $N_e = N_{O_2^+} + N_{N_2^+}$, while the initial conditions correspond to a mixture of neutral oxygen and nitrogen molecules $N_{O_2}^{(0)} = 0.21 N_{air}^{(0)}, N_{N_2}^{(0)} = 0.79 N_{air}^{(0)}$, with $N_{air}^{(0)} = 2.5\times 10^{19}$ cm^{-3}.

The parameters are $n_2 = 3.2\times10^{-19}$ cm^2/W, $U_{i,O_2} = 12.063$ eV, $U_{i,N_2} = 15.576$ eV, $K_{O_2} = 8$, $K_{N_2} = 11$, $\sigma_8 = 3.7\times10^{-96}$ s^{-1}cm^{16}W^{-8}, $\sigma_{11} = 1.8\times10^{-137}$ s^{-1}cm^{22}W^{-11}, $\sigma = 5.5\times10^{-20}$ cm^2, $\tau_c = 350$ fs, and $\beta_l \equiv \sigma_l K_l \hbar\omega_0 N_l^{(0)}$.

Figure S1 A shows the results of a simulation for a 50 fs FWHM Gaussian pulse at 800 nm. The spatial phase corresponding to the axicon-focusing with a cone angle of 4.6 degrees is added to the 15 mJ input Gaussian beam with a FWHM of 1 cm. The peak intensity of the pulse grows rapidly up to 7.6×10^{13} W/cm^2, and remains fairly constant over more than 10 cm. This level is sufficient to reach single ionization of all oxygen and nitrogen molecules. The plasma density profile immediately after the pulse is homogeneous within the main lobe of the Bessel beam, justifying the use of super-Gaussian distributions for the focal line where the energy is deposited.

1.2 Modeling electron-ion recombination and heating of air before the discharge

As the 50 fs pulse with a Bessel spatial profile propagates through air, energy is deposited via optical field ionization and mostly stored initially as the kinetic energy of the quasi-free electron cloud. To evaluate the evolution of the electron density following the arrival of the laser pulse, while a constant electric field is applied between the electrodes, we modified Eqs. (2a,b) to account for avalanche ionization and collisional processes leading to i) field-induced attachment or detachment of electrons and ii) recombination. We followed the model proposed by Zhao et al. *(40)*, considering positive ions O_2^+ and N_2^+ as a single species of density N_+, negative ions as a single species of density N_- colliding with electrons of density N_e:

$$\partial_t N_+ = \alpha N_e - \beta_{e+} N_e N_+ - \beta_{+-} N_+ N_-$$
$$\partial_t N_- = \eta N_e - \beta_{+-} N_+ N_- - \gamma N_- \qquad \text{(3 a, b, c)}$$
$$\partial_t N_e = (\alpha - \eta) N_e - \beta_{e+} N_e N_+ + \gamma N_-$$

Avalanche ionization occurs because of the collisions of electrons, accelerated in the external electric field, with neutral molecules. It is described by the rate $\alpha(N, E_e)$, which depends on the density of neutral molecules N and external electric field E_e [Eq. (43) in *(40)*]. Two and three body dissociative attachment reactions are considered, producing in dry air negative ions O_2^-, and O^- with a total rate $\eta(\rho, E_e)$ [Eqs. (46), (47) in *(40)*]. A single averaged recombination coefficient for N_2^+ and O_2^+ is considered $\beta_{e+} = \beta_{+-} = 0.79\beta_{e-N_2^+} + 0.21\beta_{e-O_2^+}$ where $\beta_{e-N_2^+}$ and $\beta_{e-O_2^+}$ are given by Eqs. (49) and (50) in *(40)*, respectively. Finally, the detachment of electrons, due to the collisions of negative ions with neutral molecules, is described by the rate $\gamma(\rho, E_e)$ [Eq. (55) in *(40)*]. As seen from Fig. S2, Eqs. (3) predicts that fractions of a nanosecond after the pulse transit, electron-ion recombination nearly neutralizes the medium.

1.3 Modeling gas dynamics

Numerical simulations of the hydrodynamic expansion of heated air are carried out by solving the compressible fluid Euler equations in two-dimensional cylindrical coordinates (neglecting the azimuthal dynamics):

$$\partial_t U + \vec{\nabla} F = S,\ U = [\rho, \rho\vec{u}, e],\ F = [\rho\vec{u}, \vec{u} \otimes \rho\vec{u} + p\hat{\mathrm{I}}, \vec{u}(e+p)],\ S = [0, \vec{0}, \vec{\nabla}(q\vec{\nabla}T)], \qquad (4)$$

where ρ is the mass density, $\vec{u}$ is the fluid bulk velocity, *p* is the pressure, *e* is the total energy density, *T* is the temperature and q=0.024 W $m^{-1}K^{-1}$ is the heat conductivity, which sets the lifetime of the low density region to the sub millisecond scale *(41)*, much

larger than the laps of time we had between the pulse transit and the onset of the discharge (see main text). Here $\otimes$ denotes a tensor product and $\hat{\mathrm{I}}$, the 2x2 identity matrix. The integration of the fluid equations is carried out through the Harten Lax van Leer first order Godunov method with the restoration of the contact surface *(35)*. This method is able to capture the shock wave formation, whose threshold is well below the peak temperatures used here, T=50,000 K. The system of equations is closed by using the equation of state and internal energy in the ideal gas forms, $p = \rho RT$ and $\varepsilon = e/\rho - u^2/2 = RT/(\gamma - 1)$, respectively, where *R*=287.058 J kg^{-1} K^{-1} and γ=1.4 are used to compute the speed of sound, $a = \sqrt{\partial_\rho p|_\varepsilon + p/\rho^2\, \partial_\varepsilon p|_\rho}$, necessary to evaluate the fluxes, *F*. Our simulations are initialized with a temperature distribution having the shape of the plasma volume generated by the first lobe of the Bessel function J_0 (interrupted by the stopper and self-healed), where the energy is deposited (see Fig. 2 and the fluorescence trace in Fig. 3C of the main text):

$$T(r,z,t=0) = T_\infty + \Delta T \cdot J_0(\kappa r)\left[\Theta(z_{obs} - z)e^{-\left(\frac{z-z_{obs}}{z_w}\right)^{2m}} + e^{-\left(\frac{z-z_{obs}-z_w-z_{sh}}{z_w}\right)^{2m}}\right], \tag{5}$$

where T_∞=300 K and ΔT=50,000 K are the background and peak temperatures, $\kappa \approx$ 2.4/(7.6×0.35)/2 μm^{-1} is related to the zero-to-zero distance of the first Bessel lobe (≈7.6 μm), and Θ(z) is the Heaviside step function. z_{obs}≈3.5 cm, z_w=3 cm and z_{sh}=1 cm are, respectively, the obstacle position, the width of the super-Gaussian distributions (with m=10), and the self-healing distance of the Bessel beam after hitting the obstacle. The latter is estimated from the obstacle height h≈0.1 cm, and the cone angle, θ≈4.6×π/180,

as z_{sh}=h/tg θ ≈1 cm (consistent with Fig. 3C in the main text). Heating of air (plasma recombination) occurs over the typical molecular collision time (~0.1 ns) at room temperature, as shown in Fig. S2, an extremely short timescale compared to both the acoustic relaxation and thermal diffusion timescales. We therefore assume that the heating of air occurs almost instantaneously after the laser pulse and does not change the gas density appreciably (since there is no time for significant molecular diffusion). Under this assumption the pressure, p=p(ρ,T), and total energy, e=e(ρ,T), are univocally defined and set the initial conditions.

1.4 Estimating the heating of air

The value of the peak temperature, ΔT, was estimated from experimental observations as follows. When the Bessel beam propagates between the two electrodes, without any obstacle, the breakup voltage, V_B (onset of the discharge), was seen to be an order of magnitude lower than that of the air at room temperature. According to the Paschen law $V_B \propto \rho$ and therefore the density of the heated air should also decrease by an order of magnitude with respect to the case of unheated air. Therefore, a series of numerical resolutions of Eqs. (4) was launched with ΔT as the only fitting parameter. The axial air density versus time, ρ(t,r=0;ΔT), was recorded until the minimum density was found to reach the indirectly observed value. This led to the conclusion that ΔT≈50,000 K. Other experimental works with Gaussian beams and similar pulse energy and duration *(41)* have used a similar procedure to estimate the variation of the peak temperatures for heated air at ΔT~10^3 K, being apparently inconsistent with our findings. However, the Bessel beam deposits energy over an air column whose cross section diameter is about 7 times smaller than that of the Gaussian beam (see main text), and hence the heated cross

section is ~50 times smaller. In addition, the presence of the external electric field, leading to Joule heating while the electron plasma recombines, is also responsible for an increase of the temperature associated to the thin column of air. Therefore, the high peak temperature values obtained here are consistent with this work and the previous literature.

1.5 Modeling the discharge path

In order to model the discharge evolution between the two electrodes, we adopted a stochastic model similar to that of Niemeyer et al. *(37)*. In this model, space is segmented in a number of cells and at each time step a new space segment is added to the existing continuous discharge pattern with an associated probability $P_i = (E_i)^\eta / \sum_j (E_j)^\eta$. In this expression, E_i is the electric field associated with the ith possible increment of the discharge pattern and η is an exponent appropriate to the specific dielectric medium and the number of dimensions considered. The new discharge increment i is determined by sorting a random number, taking into account all the probabilities P_i at a given time step.

Since the electron avalanche process that permits discharge propagation actually depends on E/N, we made the substitution $E_i \rightarrow E_i / N_i$ in the above expression for P_i, where N_i is the local air density as given by the calculation described in Sec. 1.4. The electric field was computed by solving $\nabla \cdot (\varepsilon \nabla \varphi) = 0$ numerically, where φ is the local potential ($\vec{E} = -\nabla \varphi$) and ε is the local relative permittivity (ε = 1 in air and plasma, and ε = 5 in the obstacle). The finite difference form of the latter equation for φ was solved iteratively without convergence acceleration. We used Neumann boundary conditions on the sides of the simulation box and Dirichlet conditions on the electrodes. The calculations were done in the z-y plane on a 200×100 grid. We assumed that the discharge pattern was

almost a perfect conductor with a small potential drop from the starting electrode in function of length (much less than 1% per unit length). The possible discharge increments i were selected by considering only the horizontal and vertical directions of the grid and by requiring that E_i / N_i exceeds the known breakdown threshold value. We found that the exponent η = 5 provides realistic-looking discharge patterns for both the Gaussian beam (Fig. 2B) and the quenched Bessel beam (Figs. 3D and 4). The simulation stops when no more increments are available (mainly due to below-threshold values of E_i / N_i for all i) or when the two electrodes are connected. Since the discharge can in principle grow from both electrodes, we assumed that two discharge branches grow simultaneously from both electrodes with equal velocity. The high electric field generated when the two branches get close to each other implies that they generally connect smoothly, thus forming a continuous discharge pattern between the two electrodes.

2. Experimental setting

Differently from a large part of the literature, where discharge guiding was addressed over meters or even longer distances, here we have investigated the dynamics of electric discharges occurring on only 1-10 cm scale. This is a typical distance that may be relevant for e.g. high-voltage switching (spark gap) or for machining, as indeed only few centimeters long discharges are employed for cutting and for material processing. Given the air breakdown field of nearly 34 kV/cm, and having set a 5 cm distance between the electrodes, it follows that the high voltage source must not exceed 170 kV. We employed a high-voltage source, able to deliver up to 35 kV DC, to bias the two wire electrodes at a field much lower that the spontaneous breakdown in air. In order to initiate a discharge

we are therefore required to consistently lower the breakdown field by at least an order of magnitude. The laser source available at the Advanced Laser Light Source (at INRS-EMT campus, Varennes, Canada) was a 100 Hz repetition rate, 100 mJ energy, 50 fs full-width at half-maximum pulse duration, Ti:Sapphire amplified system (Thales, France) with a Gaussian spatial output profile of nearly 10 mm full-width at half maximum. We observed that in all the focusing conditions employed in our measurements, 20 mJ of input energy were sufficient to trigger the discharge process.

2.1 Gaussian beam

The first experiment consisted in generating a laser-induced plasma between the two electrodes by focusing the output Gaussian beam with a plano-convex, f=100 cm, fused silica lens. This is the most common approach to trigger a short-range electric discharge. To set a working condition that can be compared to what happens with different beam profiles, we characterized the bias voltage required to drive a discharge over 4 cm in function of the energy of the laser pulse and we reported the results in terms of the ratio between the required breakdown field and the air breakdown field (BD field) in function of the laser energy. We have observed that for energy as high as 14 mJ, the breakdown field reduces to 25 % of the standard air value, as shown in Fig. S3 A. It is relevant to note that the breakdown process shows a clearly stochastic spatial nature, as can be appreciated by the segmented shape of the electric discharge recorded in a single shot and shown in Fig. 2B (manuscript text). Different discharges show a different pattern. We relate this stochastic nature to the fact that the conductive channel generated by the thermalization of the laser-induced plasma has a transverse dimensions of the order of

100 μm and the discharge can hence choose any of the low resistance paths within the conducting volume.

2.2 Self-bending beam

In the second experiment we employed a self-bending beam generated by exploiting the third order phase aberration induced by a tilted cylindrical lens with a 40 cm focal length [see e.g. *(22)*]. This generates a 1D self-bending beam while in the other dimension we employed another cylindrical lens (60 cm focal length, not aberrated) to focus the beam. This way we observed the generation of a curved plasma channel with a radius of curvature of nearly 55 cm. The choice of exploiting the coma aberration is dictated by the ease of its realization at high energy, as in this case no phase masks or specialized optics is required and the result well serve the purpose of illustrating the effect. Other, more sophisticated means of creating self-bending beams can be employed, as, e.g., we have realized a similar self-bending beam by means of a binary phase mask (fabricated via laser lithography on a layer of chromium sputtered on a glass substrate). The design was meant to generate a concatenation of Airy beams and we have employed it for demonstrating the possibility of S-shaped controlled electric discharges (see Fig. 2G and H in the main text). For details on the generation of Airy beams by means of binary phase masks we refer to *(42)*.

We have characterized the reduction of the breakdown voltage in case of a self-bending beam generated by the aberrated cylindrical lens system and the results are shown in Fig. S3 A. For energies below 8 mJ, we were not able to observe the discharge for the range of potentials provided by the high-voltage source.

2.3 Bessel beam

In the third experiment we used an apodized Bessel beam to generate a plasma filament between the electrodes. We produced the finite energy Bessel beam by employing a fused silica conical lens (axicon) with a 160 deg base angle, producing a Gaussian apodized Bessel with a cone angle of 4.6 degrees. The transverse spatial profile of this wavepacket is characterized by a narrow peak with a zero-zero distance of 7.6 μm, hence much more localized in space that the Gaussian counterpart. The input beam profile allows for several centimeters of non-diffractive propagation (nearly 9 cm full-width at half maximum for the central peak intensity, considering an 11.3 mm full-width at half maximum Gaussian apodization). Even in this case we have characterized the breakdown voltage/energy characteristic at a fixed electrodes distance of 4 cm and the results are reported in Fig. S3 A. Remarkably, the Bessel beam case allows discharges with a field that is as low as the 10% of the air breakdown counterpart. Furthermore, the stochastic behavior featuring the Gaussian case is here completely eliminated in virtue of a much narrower and spatially selective low resistance channel.

The Bessel beam case is clearly the most advantageous in terms of breakdown voltage reduction and we have further evaluated the relation between the applied voltage and the distance between the electrodes. The results are shown in Fig. S3 B and clearly highlight a linear dependence of the required bias voltage from the electrodes distance which hint, in turn, toward a uniform highly conductive channel triggered by the laser irradiation. We have observed that with 12 mJ of input pump power and nearly 33 kV of applied voltage we are able to produce a very uniform electric discharge over more that 9 cm (see Fig. S3 C and D).

2.4 Self-healing with a metallic obstacle

The self-healing experiments reported in the main manuscript have been performed employing a high optical density dielectric stopper. The question arises on the effect of a metallic stopper. We have performed an additional experiment employing a metallic stopper placed on an isolated post. The result of an acquisition over multiple discharge events is shown in Fig. S3 E. It is clear from the figure that the electric discharge hits the metallic obstacle and restarts from it over random paths, finally converging to a well-defined trajectory once the self-healing process rebuilds the ionized channel along the pulse propagation direction.

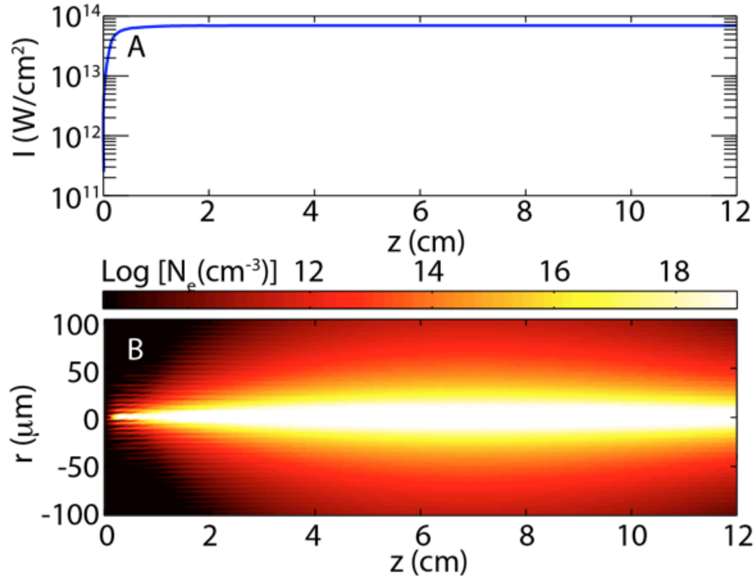

Fig. S1.

Pulse propagation simulations. (**A**) Peak intensity on the propagation axis and (**B**) plasma channel density obtained by numerical simulation for a 15 mJ, 800 nm, 50 fs pulse focused in air with an axicon. The Bessel cone angle is 4.6 deg. The color bar shows $\log_{10}$ [N_e(cm^{-3})].

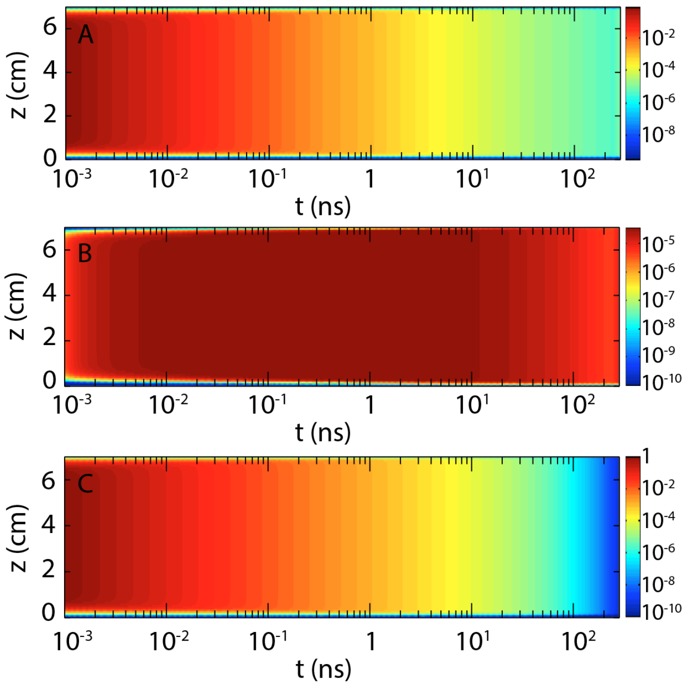

Fig. S2

Density profiles. Evolution of the density profile over 250 ns following the arrival of the laser pulse, and calculated by numerical simulation of Eqs. 3 (a, b, c) in air at 1 bar for an applied electric field of 2 kV/cm. (**A**) Density N_+ of positive ions. (**B**) Density N_- of negative ions. (**C**) Density N_e of free electrons. The colorbars refer to the fraction of atmospheric density 2.5×10^{19} cm^{-3}.

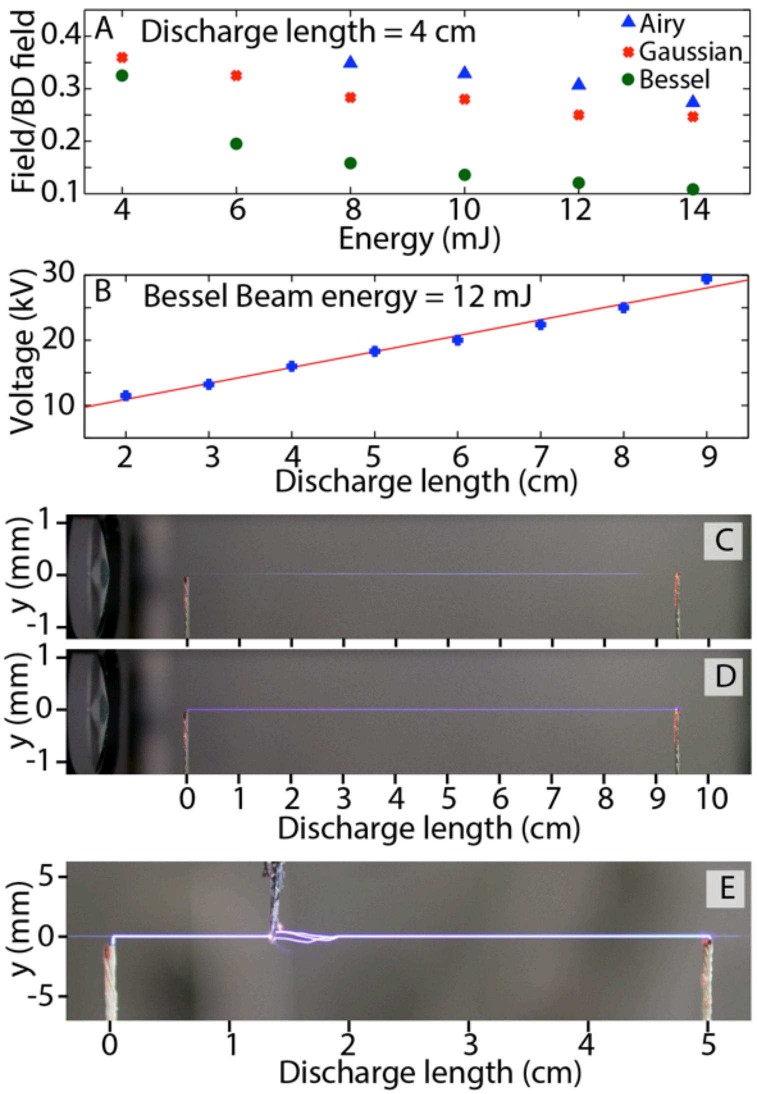

Fig. S3

Additional experimental results. (**A**) Energy dependent reduction of the breakdown field for the three beam profiles employed in the experiments and for a fixed electrodes distance of 4 cm. (**B**) Electrode distance dependence of the required voltage for observing a breakdown in the case of a Bessel beam profile and for a 12 mJ pulse energy. (**C**) Ionized channel generated by a 12 mJ Bessel beam. (**D**) Electric discharge triggered by the condition in (**C**) and for an applied voltage of nearly 33 kV. (**E**) Multi-shot acquisition of different discharge events in case of a metallic obstacle partially blocking the intense ionized channel generated by a Bessel beam.
